# Expression pattern and prognostic value of key regulators for N7-methylguanosine RNA modification in prostate cancer: m7G RNA modification in PCa

**DOI:** 10.3724/abbs.2023017

**Published:** 2023-02-22

**Authors:** Qiliang Zhai, Yan Hou, Yuedian Ye, Sujuan Dai, Guangxiu Guo, Qiao Yang, Guofu Pang, Qiang Wei

**Affiliations:** 1 Department of Urology Nanfang Hospital Southern Medical University Guangzhou 510515 China; 2 Department of Urology Ganzhou People’s Hospital Ganzhou 341000 China; 3 Department of Anesthesiology Nanfang Hospital Southern Medical University Guangzhou 510515 China; 4Department of Pathology Ganzhou People′s Hospital Ganzhou 341000 China; 5 Department of Urology Zhuhai People’s Hospital (Zhuhai Hospital affiliated with Jinan University) Zhuhai 519000 China

**Keywords:** prostate cancer, m7G, RNA modification, tumor microenvironment, tissue microarray

## Abstract

Alterations in the regulators of RNA methylation modifications, such as N7-methylguanosine (m7G), have been implicated in a variety of diseases. Therefore, the analysis and identification of disease-related m7G modification regulators will accelerate advances in understanding disease pathogenesis. However, the implications of alterations in the regulators of m7G modifications remain poorly understood in prostate adenocarcinoma. In the present study, we analyze the expression patterns of 29 m7G RNA modification regulators in prostate adenocarcinoma using The Cancer Genome Atlas (TCGA) and perform consistent clustering analysis of differentially expressed genes (DEGs). We find that 18 m7G-related genes are differentially expressed in tumor and normal tissues. In different cluster subgroups, DEGs are mainly enriched in tumorigenesis and tumor development. Furthermore, immune analyses demonstrate that patients in cluster 1 have significantly higher scores for stromal and immune cells, such as B cells, T cells, and macrophages. Then, a TCGA-related risk model is developed and successfully validated using a Gene Expression Omnibus external dataset. Two genes ( *EIF4A1* and *NCBP2*) are determined to be prognostically significant. Most importantly, we construct tissue microarrays from 26 tumor specimens and 20 normal specimens, and further confirm that EIF4A1 and NCBP2 are associated with tumor progression and Gleason score. Therefore, we conclude that the m7G RNA methylation regulators may be involved in the poor prognosis of patients with prostate adenocarcinoma. The results of this study may provide support for exploring the underlying molecular mechanisms of m7G regulators, especially EIF4A1 and NCBP2.

## Introduction

Currently, prostate cancer (PCa) is one of the most common malignancies of the male urological system. According to cancer statistics, there were approximately 248,530 new cases of PCa and 34,130 deaths in the United States in 2021, accounting for 26% of all malignancies and 11% of cancer mortalities [1]. Most patients diagnosed with localized PCa recover with standard treatments, such as radical prostatectomy or radiation therapy [2]. However, biochemical recurrence (BCR) is common, occurring in approximately 35% of patients who have undergone treatment for localized PCa [3]. In patients with higher Gleason scores and rapid prostate-specific antigen (PSA) elevations, BCR is associated with poorer survival [4]. Most patients with BCR develop clinical recurrence and metastasis, eventually leading to death. Although there are many prognostic indicators for patients with PCa, such as the Gleason score and PSA, they have limited ability to predict the timing of a patient’s BCR [5]. Therefore, further development of novel biomarkers with strong specificity and high accuracy is crucial for predicting the prognosis of localized PCa and guiding the treatment of these patients.

There is growing evidence that the proteins responsible for RNA modification participate in the development of human disease. RNA modification plays a key role in many cellular processes, as does the methylation of guanosine to form N7-methylguanosine (m7G) [ 6, 7] . It is one of the most conserved modified nucleosides and is commonly found in eubacteria, eukaryotes [8], and some archaea [9]. In the 5′ cap of the mRNA, abundant m7G methylation modification can improve mRNA stability and regulate many important biological mechanisms, such as mRNA transcription, pre-mRNA splicing, 3′ polyadenylation, cell nuclear export, and mRNA translation [ 10– 13] . In addition, m7G has been shown to be widely present in transfer RNA (tRNA) and 18S ribosomal RNA (rRNA), which play an important role in maintaining RNA processing, metabolism, stability, and nucleation, as well as protein translation [ 14– 16] . Additionally, alterations in the regulators of m7G modifications have been implicated in a variety of diseases. Specifically, recent studies have reported that *METTL1*-mediated m7G methylation maintains the pluripotency of human stem cells and limits vascular development [11]. Furthermore, *METTL1* has been reported to affect the viability of cancer cells [17], and *WDR4* overexpression affects learning and memory in patients with Down syndrome [18]. However, the disease-related m7G modification regulators remain poorly characterized in PCa. Therefore, the analysis and identification of disease-related and m7G modification-related regulatory genes will accelerate advances in understanding disease pathogenesis at the molecular level and the in-depth evaluation and treatment of the prognostic risks of various cancers.

However, exploring the links between m7G-regulated genes and various diseases through basic experiments alone can be time-consuming and expensive. Herein, we aimed to analyze the expression patterns of 29 m7G RNA modification regulators in prostate adenocarcinoma (PRAD) using The Cancer Genome Atlas (TCGA) and to assess the immune infiltration status and pathway enrichment of genes differentially expressed in different subgroups. In addition, a TCGA-related risk model was developed and successfully validated using the Gene Expression Omnibus (GEO) external dataset. Then, we established a tissue microarray (TMA) using 26 cancerous tissue samples and 20 normal tissue specimens to further confirm the expressions of m7G regulators using immunohistochemistry (IHC). We further confirmed that the expressions of EIF4A1 and NCBP2 were associated with cancer progression. Our study may help explore the underlying molecular mechanisms of m7G regulators in PRAD and shed light on appropriate targeting therapy strategies for patients with PRAD.

## Materials and Methods

### Data collection

TCGA-PRAD data were gathered from the UCSC Xena browser ( http://xena.ucsc.edu/). They included gene expression datasets (RNA-seq) on patients with PRAD, as well as the corresponding demographic (age and sex), clinicopathological (pathologic T stage, pathologic N stage, and Gleason score), and survival [progression-free survival (PFS)] information. Samples acquired from the GEO database (GSE21034; http://www.ncbi.nlm.nih.gov/geo) were included and defined as the verification cohort after integration, which contained 140 PRAD samples with corresponding gene expression data. The clinicopathological and survival (BCR-free survival) information for GSE21034 was obtained from cBioPortal ( www.cbioportal.org). Patients without survival information were excluded from further evaluation. Then, 29 m7G RNA modification regulators were manually selected from the literature [19] and the Molecular Signatures Database (MsigDB; https://www.gsea-msigdb.org/gsea/msigdb/index.jsp). The raw data were integrated, and the results of the site database analysis were validated using R 4.1.2 and Sangerbox 3.0 tools ( http://www.sangerbox.com/tool). Sangerbox is an online analysis tool based on R software and R packages that has been widely used and validated [ 20– 22] .

### TMA construction and patient information

This study conformed to the ethical guidelines of the Declaration of Helsinki and was approved by the Ethics Committee at the Nanfang Hospital of Southern Medical University (No. NFEC-2022-229). All study participants provided informed consent. A total of 26 cancerous and 20 normal tissue samples from patients who underwent radical prostatectomy in the Department of Urology, Nanfang Hospital, from 2019 to 2021 were identified. None of the patients enrolled had received hormone therapy or preoperative radiotherapy. The original hematoxylin and eosin-stained prostatectomy specimen slides were evaluated by two pathologists. The clinical characteristics and pathological data were extracted and collected from the medical records and pathology reports of enrolled patients. It was confirmed that each specimen represented a diagnostic region. Two cores (1 mm in diameter) were separately selected, transferred uniformly to the receptor block, and cut into consecutive 3-mm sections to make the TMA.

### IHC and scoring

We used a eukaryotic translation initiation factor 4A1 (EIF4A1) antibody (#250072; dilution 1:1200; ZenBio, Chengdu, China) and nuclear cap-binding protein subunit 2 (NCBP2) antibody (#GB112277; dilution 1:300; Servicebio, Wuhan, China) to perform IHC on the TMA sections. The stained slides were scanned using a NanoZoomer S360 (Hamamatsu Photonics K. K, Hamamatsu, Japan) and observed using NDP.view 2 Plus software (Hamamatsu Photonics K. K).

The TMA-IHC results were scored as follows according to the stain intensity of the positive marker (0–3): dark brown staining, strong (score, 3); light brown staining, moderate (score, 2); light yellow staining, weak (score, 1); and no staining, negative (score, 0). According to the percentage of positive cells (0–4): 4, 76%–100%; 3, 51%–75%; 2, 26%–50%; 1, 6%–25%; and 0, 0–5%. The TMA-IHC score was the product of both metrics. Two independent pathologists blinded to the specimen information scored the slides. When their opinions were inconsistent, a third pathologist who was also blinded to the specimen information was asked to give the final score.

### m7G modification regulator expression level analysis

Sangerbox tools were used to draw an expression heatmap of the m7G modification regulators for TCGA-PRAD data. The violin plot and correlation heatmap were plotted using Hiplot (version 0.1.0; https://hiplot.com.cn/basic/violin-group?lang=en). Then, GeneMANIA ( http://genemania.org/search/) was used to construct a gene–gene interaction network for differentially expressed genes (DEGs) to evaluate gene functions.

### Identification of the molecular subgroups

A cluster analysis was performed using the R Software package “ConsensusClusterPlus” and aggregated kmdist clusters with 1-Pearson correlation distances and resampling 80% of the samples for 10 repetitions. For the principal component analysis (PCA), we used the R package “stats.”

### Functional analyses

The DEGs between the two clusters were obtained using the R package “Limma”. Then, we used the R package “org.hs.eg.db” for Gene Ontology (GO) annotation of differentially upregulated genes, which was used as the background set, following which we mapped genes to the background set. The enrichment analysis was performed using the R package “clusterProfiler” to obtain the gene set enrichment results. We obtained the latest gene annotations of the Kyoto Encyclopedia of Genes and Genomes (KEGG) pathway using the KEGG REST API database ( https://www.kegg.jp/kegg/rest/keggapi.html) as the background, mapping genes to the background set, and using the R software “clusterProfiler” for enrichment analysis. Metascape ( https://metascape.org/) was used for protein–protein interaction enrichment analysis of differentially upregulated genes. We performed gene set enrichment analysis (GSEA) using GSEA software (version 3.0). Then, the GSEA results were presented using the Sangerbox tool. Samples were divided into two groups based on the clustering results. The “c2.cp.kegg.v7.4.symbols.gmt” and “c5.go.bp.v7.4.symbols.gmt” subsets were downloaded from the MsigDB to assess the relevant pathways and molecular mechanisms.

### Immune analyses

The “IOBR” is a computing tool for immuno-tumor biological research [23]. Here, we used the R software package “IOBR,” selecting the ESTIMATE, TIMER, and MCP-counter methods to calculate the score of the infiltrating immune cells in each sample and plot. Statistical analyses were conducted according to the different groups.

### Establishment and validation of the risk model

We used the R software package “Survival” to integrate the survival status and gene expression data and screened eight genes with prognostic significance. Subsequently, we performed a regression analysis using the R package “glmnet” [using the least absolute shrinkage and selection operator (LASSO) analysis method]. In addition, we set a 10-fold cross-validation to obtain the optimal model. When the lambda value was set to 0.0528580442917538, we finally obtained two genes. The risk scores were calculated as follows: risk score=0.204561078224938* *EIF4A1*+ 0.0313509342771888* *NCBP2*. Then, Kaplan–Meier curves were constructed, and a log-rank test was used to evaluate the survival differences between the high- and low-risk groups. Additionally, receiver operating characteristic (ROC) analysis was performed using the R package “pROC” to obtain the area under the curve. The sensitivity and specificity of the risk model were assessed using ROC curve analysis.

### Statistical analyses

**Figure 1:**
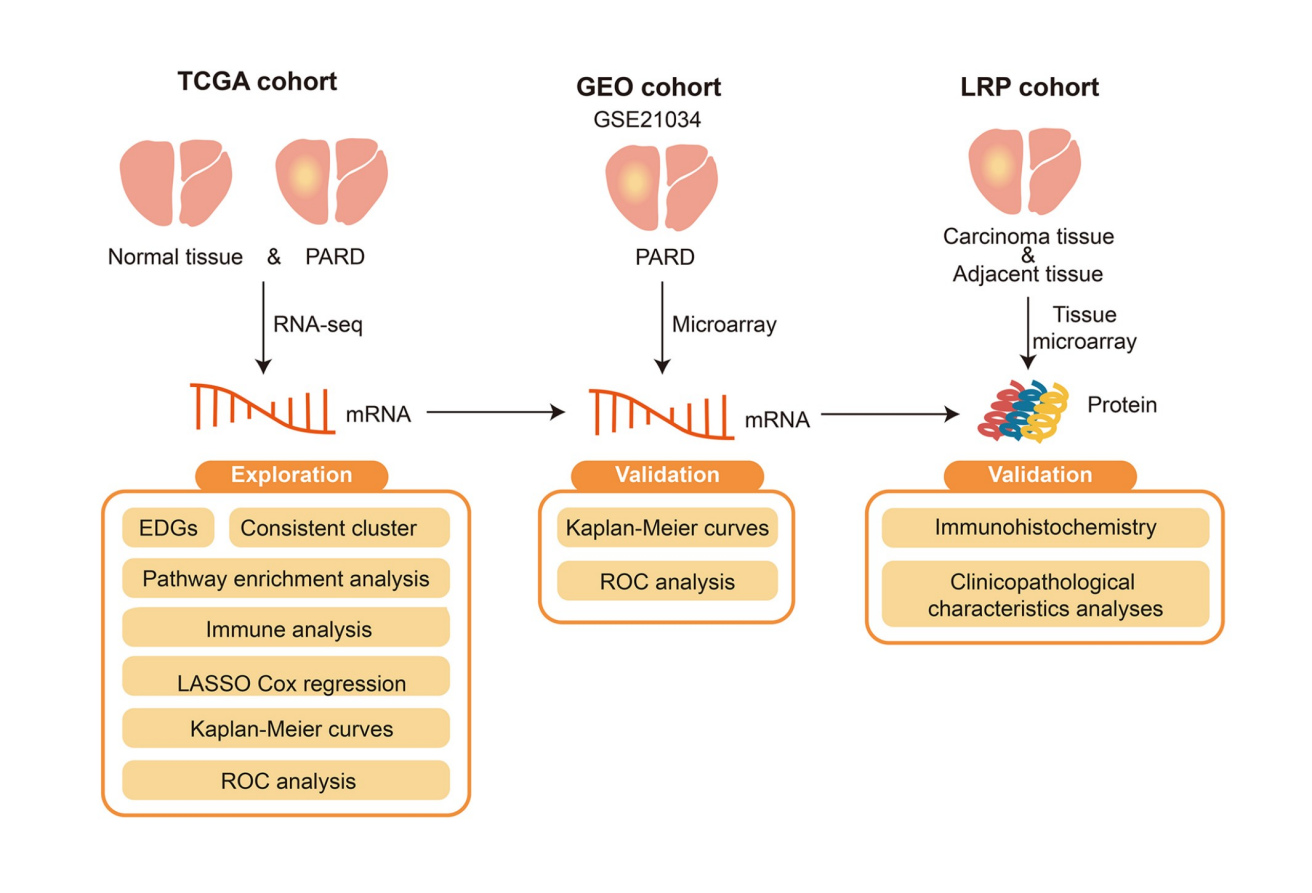
Figure 1 The overall analysis flow chart of this study

Statistical analysis of all data was conducted using the R packages as described above. In general, discontinuous data are shown as numbers or percentages, and continuous data are expressed as the mean±standard deviation (SD). Student’s *t* test was used for statistical analysis between two groups, and one-way analysis of variance was selected to compare multiple groups. A *P*<0.05 was defined as the threshold for statistical significance. The overall data analysis rationale of this study is shown in Figure 1.

## Results

### Gene expression of the m7G RNA modification regulators jointly involved in PCa development

**Figure 2:**
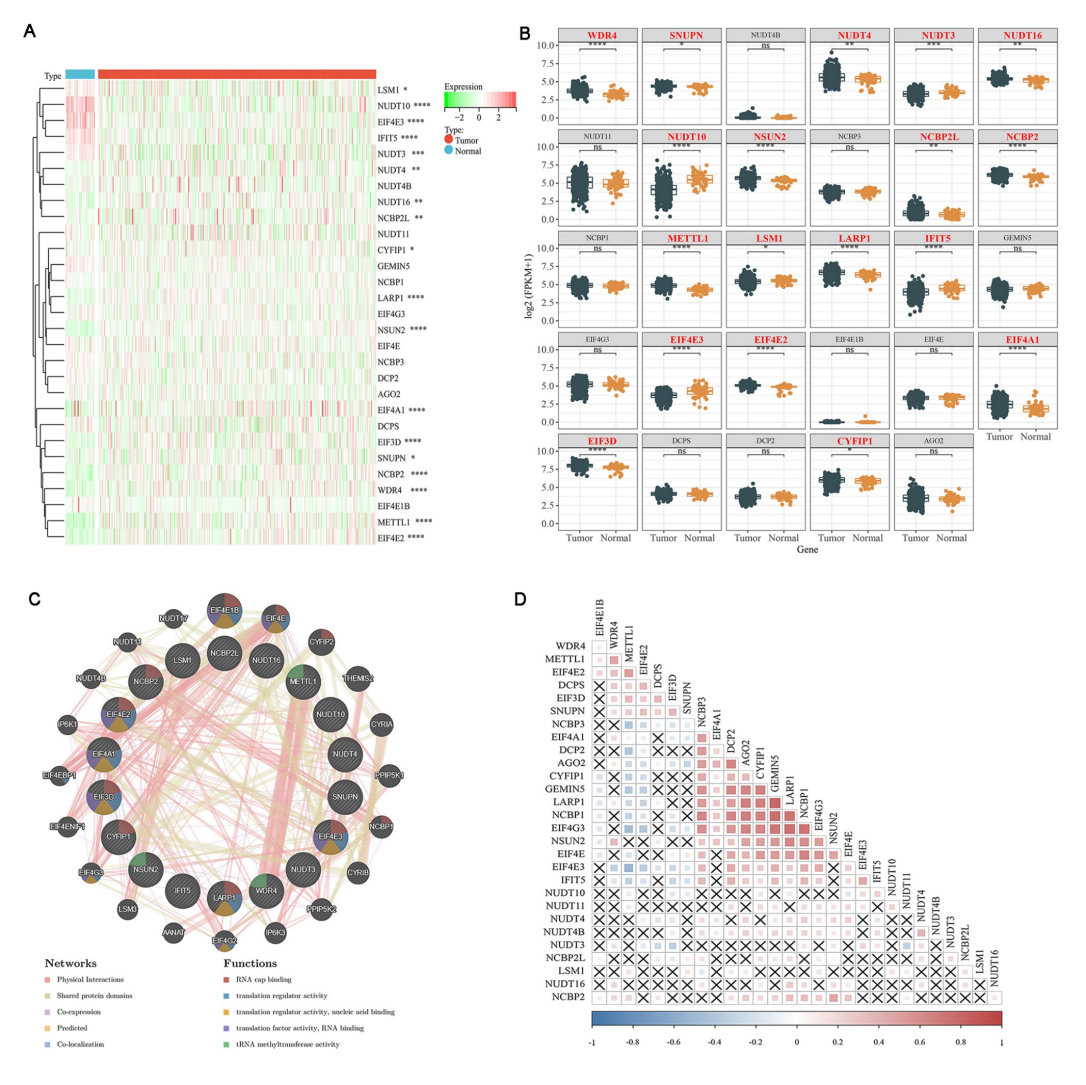
Figure 2 Expression of m7G modification regulators in prostate adenocarcinoma (PRAD) (A) The heatmap visualizes the expression levels of m7G RNA modification regulators in each sample of TCGA-PRAD. They were classified as normal and tumor samples. Green represents low expression, and red represents high expression. (B) The box plot shows the differentially expressed m7G RNA modification regulators in PRAD. They were classified as normal and tumor samples. (C) The gene‒gene interaction networks of m7G-related DEGs were analyzed by the GeneMANIA database. The 20 most frequently changed neighboring genes are shown. Each node represents a gene. The node color represents the possible functions of the respective gene. The connection of the node represents the expression regulation relationship between genes. (D) Spearman correlation analysis of the 29 m7G RNA modification regulators in PRAD. P>0.05 was removed directly. * P<0.05; ** P<0.01; *** P< 0.001; **** P<0.0001; ns: P>0.05.

We conducted a differential expression analysis of 29 m7G regulatory genes in PCa tissue ( *n*=499) and para-cancer tissue ( *n*=52). As shown in Figure 2A,B, 18 of the 29 m7G regulators were differentially expressed with a significance value of *P*<0.05. This included 13 upregulated and 5 downregulated genes, indicating obvious variation in m7G modification in tumorigenesis. We constructed a gene‒gene interaction network for the 18 m7G-related DEGs to analyze their function using GeneMANIA, which suggested a close connection between them. The hub node representing the DEGs was surrounded by 20 nodes representing genes that significantly correlated with DEGs ( Figure 2C). In addition, GeneMANIA analysis displayed “Networks” that were not found in GSEA. The gene connections and their colors showed that there were “physical interactions” and “shared protein domains” between most m7G-related DEGs. Moreover, a correlation analysis was performed to further understand the intrinsic association between the 29 m7G RNA modification regulators. Our analysis showed that most m7G RNA methylation regulators correlated with others. The correlation between *GEMIN5* and *LARP1* was the most significant. However, some regulators, such as *NUDT3*, *NUDT16*, *EIF4E1B*, and *LSM1*, showed poor correlation with other regulators, which may be due to their involvement in multiple biological processes.

### Consensus clustering categorizes patients based on m7G-related DEGs

**Figure 3:**
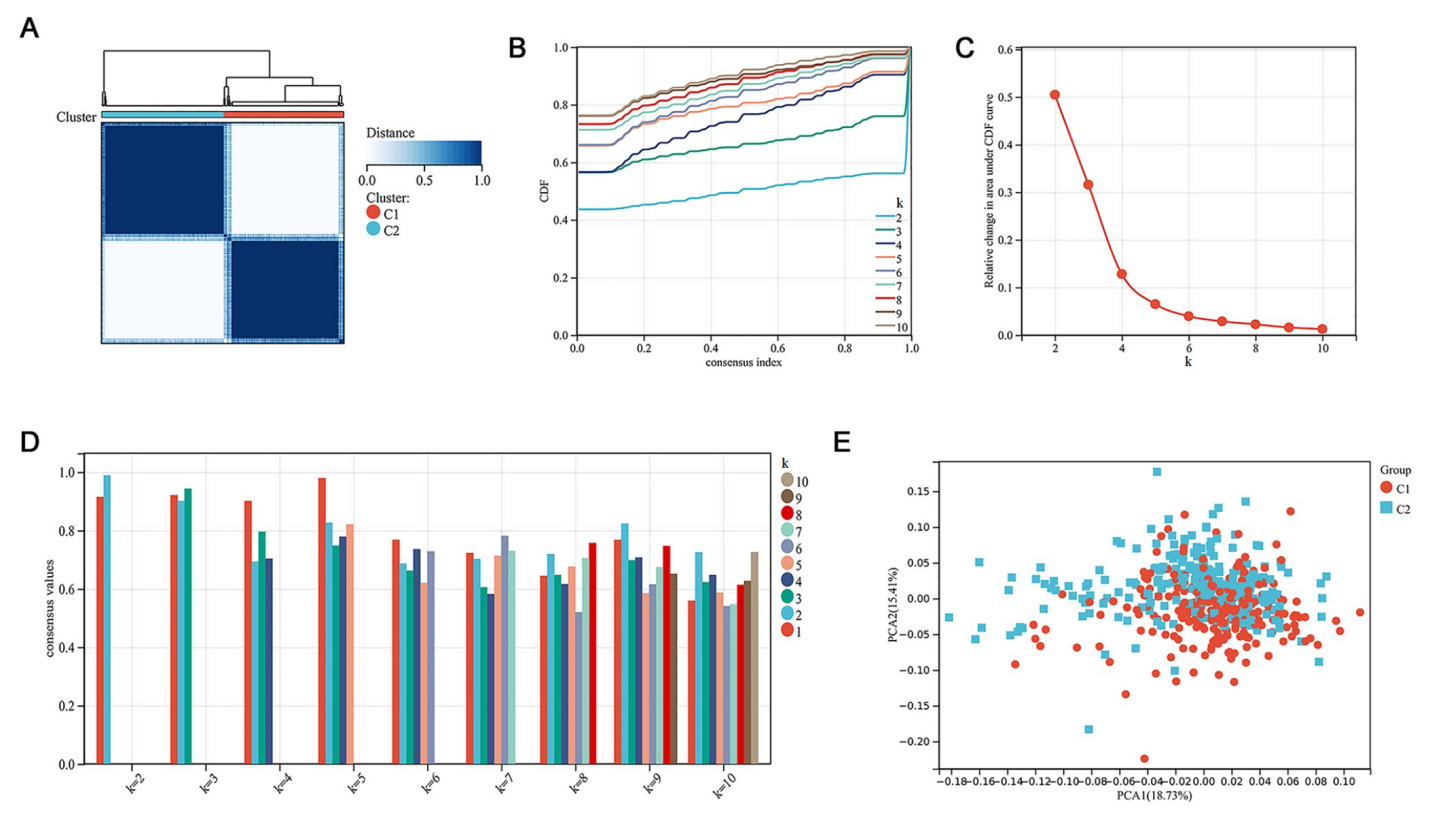
Figure 3 Consistent cluster analysis of PRAD based on the m7G-related DEGs (A) The correlation between subgroups when cluster number k=2. (B) The cumulative distribution function (CDF) is displayed for k=2–10. (C) The relative change in area under the CDF curve for k=2–10. (D) According to the evaluation of the average consistency within the sample cluster group, the number of clusters with the highest average consistency within the group is k=2, and the number of clusters with the second highest average consistency is k=3. (E) Principal component analysis (PCA) of the total transcriptomic profile from the TCGA-PRAD cohort for optimal k=2.

From the above results, the alteration of the m7G methylation regulators in PCa was confirmed transcriptomically. Therefore, it was reasonable to continue to analyze its significance in terms of its oncogenic effects. A consensus clustering approach was conducted to divide patients with PRAD into subgroups based on the RNA-seq data of the 18 m7G-related DEGs. The optimal clustering stability was identified when k=2 ( Figure 3A–D). Overall, 246 patients were clustered in cluster 1, and 250 were clustered in cluster 2. To assess the validity of the consensus clustering and offer insight into the two clusters, we performed PCA on PRAD samples. The results showed significant differences between clusters 1 and 2 ( Figure 3E).

### Patients in the two molecular subtypes exhibited different functional analyses and immune statuses

**Figure 4:**
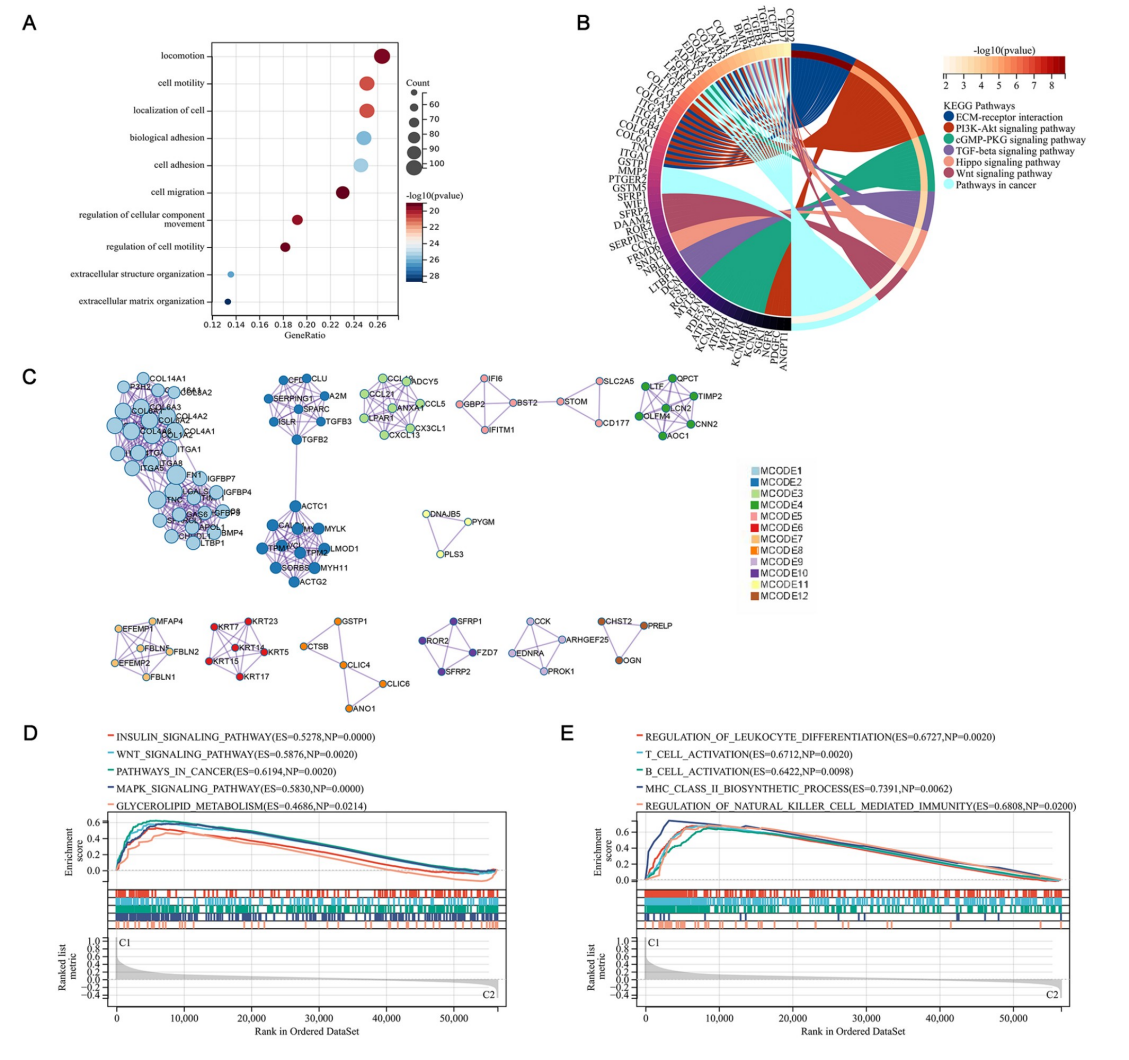
Figure 4 Differential characteristics between clusters by DEGs of two clusters (A) Gene Ontology (GO) analysis of DEGs between two clusters. (B) Kyoto Encyclopedia Genes and Genomes (KEGG) analysis of DEGs between the two clusters. (C) Protein‒protein interaction (PPI) enrichment analysis of DEGs. (D,E) GSEA plots visualizing the results of GSEA by “C2: KEGG gene sets” (D) and “C5: GO biological processes” (E). The gene sets were from MisgDB.

We identified and characterized the DEGs between the two clusters and performed functional enrichment analysis to explore their potential signaling mechanisms. A total of 443 DEGs were detected, of which 431 genes were upregulated and 12 were downregulated in cluster 2 compared to cluster 1 ( Supplementary Figure S1A). The GO enrichment analysis revealed that the DEGs were enriched in tumor motility and migration biological processes, including cell motility, biological adhesion, cell adhesion, cell migration, regulation of cellular component movement, and regulation of cell motility ( Figure 4A). Similarly, the KEGG enrichment analysis identified certain signaling pathways associated with tumorigenesis, tumor development, and some classical pathways in tumors, including the ECM-receptor interaction, PI3K-AKT signaling pathway, cGMP-PKG signaling pathway, TGF-β signaling pathway, Hippo signaling pathway, and Wnt signaling pathway ( Figure 4B). The protein–protein interaction analysis identified 12 submodels, some of which were closely associated with tumor development, glycosaminoglycan metabolism, and immunity, indicating that the immune system and metabolism may be associated with the contribution of m7G RNA methylation to PRAD ( Figure 4C). To further explore the relationship between the enriched pathways and the prognosis of patients with PRAD, we performed GSEAs to evaluate the relative expression difference in the pathways of the two clusters ( Figure 4D,E). GSEA showed many differentially expressed pathways, including classical pathways associated with tumorigenesis and development, lipid metabolism pathways, and pathways involved in immune regulation. All these results demonstrated that the expression of m7G RNA methylation regulators was correlated with the dysregulation of the immune system, metabolism, and some of the classical tumor pathways, which may be involved in the poor prognosis of patients with PRAD.

**Figure 5:**
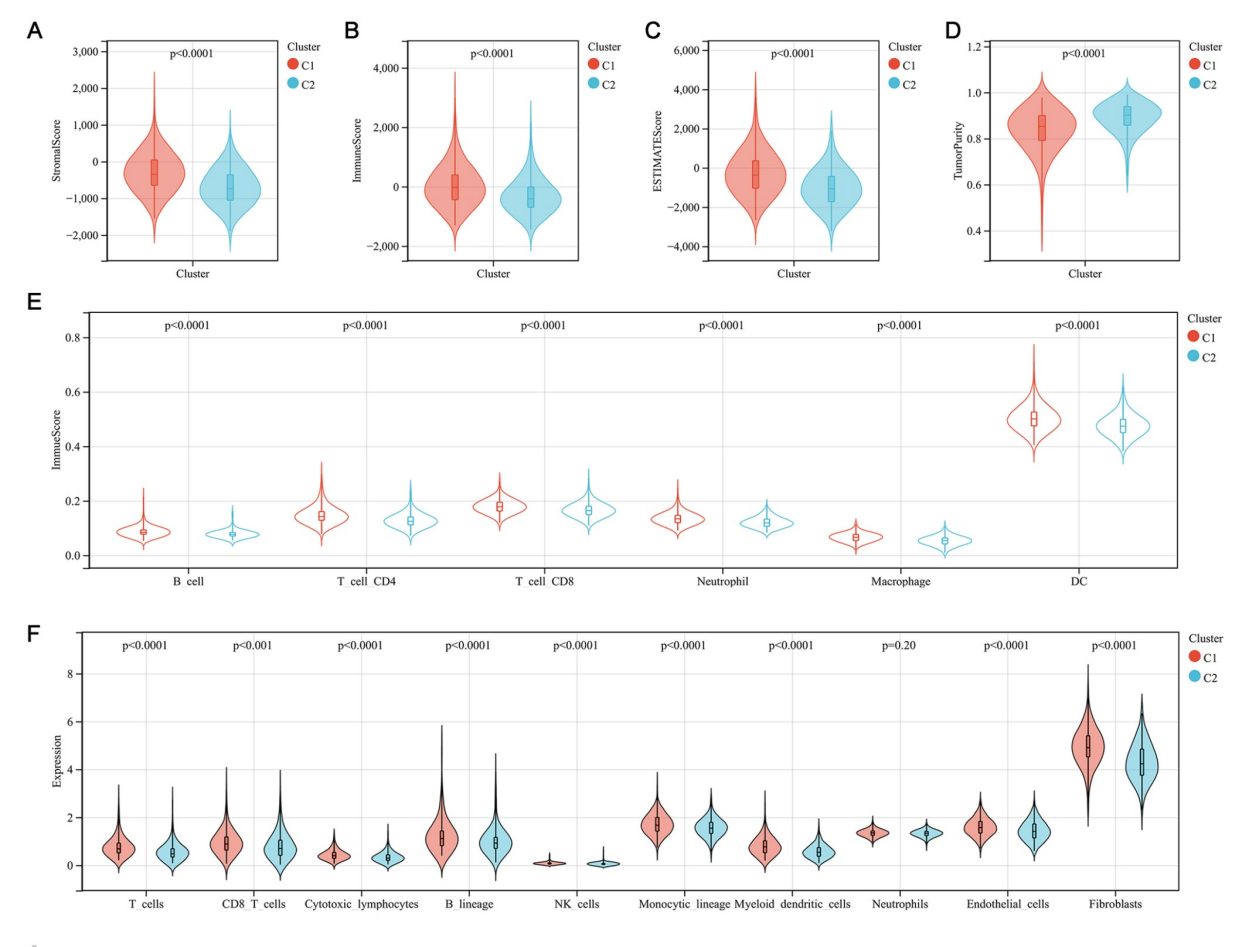
Figure 5 Immune analyses in two clustered subgroups (A) Stromal score, (B) immune score, (C) ESTIMATE score, and (D) tumor purity calculated by the ESTIMATE algorithm. (E) Abundance of 6 infiltrating immune cells evaluated by TIMER. (F) Abundance of 8 immune filtrating cells and 2 stromal cells evaluated by MCP-counter. P<0.05 was defined as statistically significant.

The composition and abundance of immune cells in the tumor microenvironment strongly influence tumor progression and the efficacy of immunotherapy. The above functional enrichment analysis indicated a significant difference in immune dysregulation between the two molecular subtypes. To explore the immune differences between the two molecular subtypes, we performed immune analysis on the transcriptomic data. The ESTIMATE algorithm revealed that patients in cluster 1 had significantly higher stromal ( *P*<0.0001), immune ( *P*<0.0001), and ESTIMATE scores ( *P*<0.0001) and lower tumor purity ( *P*<0.0001) than those in cluster 2 ( Figure 5A–D). In addition, the TIMER algorithm showed that the abundances of all types of immune cells in cluster 1 were significantly higher than those in cluster 2 ( *P*<0.0001; Figure 5E). Additionally, the infiltrating immune cell scores, calculated for each sample by the MCP-counter algorithm, showed that seven immune cells and two stromal cells were significantly higher in cluster 1 than in cluster 2, except neutrophils ( Figure 5F). These results suggest that the immune status of the two molecular subtypes is significantly different.

### Establishment and validation of a prognostic risk model based on m7G regulatory genes

**Figure 6:**
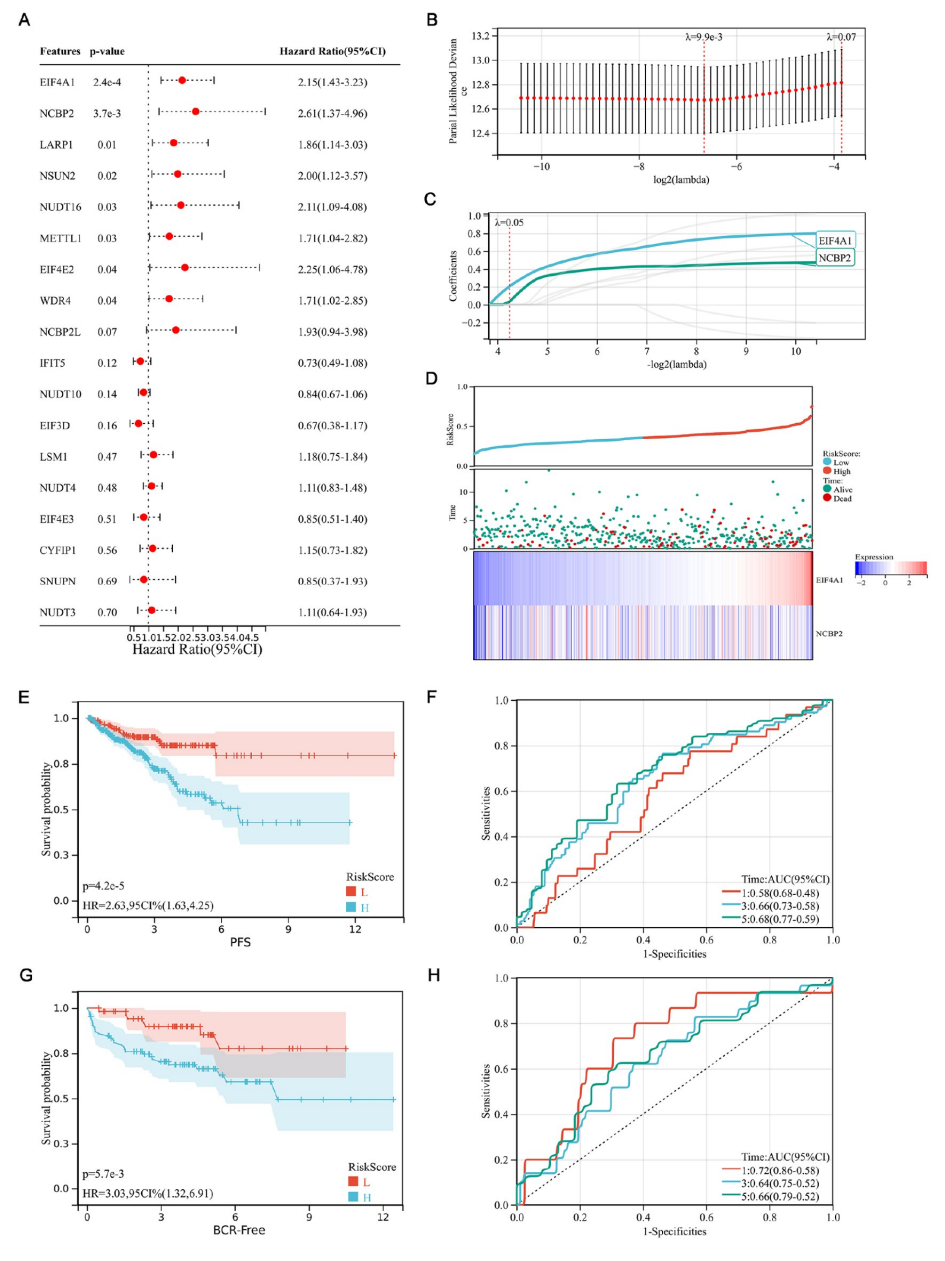
Figure 6 Risk model for PRAD patients based on m7G regulatory genes (A) Univariate Cox regression analysis revealed that the eight differentially expressed m7G methylation regulators significantly correlated with clinical prognosis. (B) The tuning parameters (logλ) of PFS-related genes were selected to cross-verify the error curve. According to the minimal criterion and 1-se criterion, perpendicular imaginary lines were drawn at the optimal value. (C) The LASSO coefficient profile of 8 PFS-related m7G methylation regulators and perpendicular imaginary lines were drawn at the value chosen by 10-fold cross-validation. (D) Distribution of survival status and risk score of PRAD patients in the high- and low-risk groups. Heatmap illustrating the expression of the two candidate genes in the two groups. (E) Survival curve of the PRAD patients in the two groups. L, low-risk group; H, high-risk group. (F) Time-dependent ROC curve of the risk model. (G) Survival curve of PRAD patients in the two groups in the verification cohort (GSE21034). L, low-risk group; H, high-risk group. (H) Time-dependent ROC curve of the risk model in the verification cohort (GSE21034). P<0.05 was defined as statistically significant.

We further explored the prognostic value of m7G regulatory genes in PRAD. We evaluated the prognostic significance of each gene using the R package “survival” and the Cox method, integrating survival time, survival status, and gene expression data. The results showed that eight of the 18 m7G-related DEGs were significantly associated with PFS ( *P*<0.05; Figure 6A). All eight regulators, namely, *EIF4A1*, *NCBP2*, *LARP1*, *NSUN2*, *NUDT16*, *METTL1*, *EIF4E2*, and *WDR4*, were considered risk genes with a hazard ratio (HR)>1. Subsequently, considering the strong association between the m7G regulators and the prognosis of patients with PRAD, we conducted a LASSO Cox regression analysis for those eight regulators to create a comprehensive and effective risk signature for prognosis ( Figure 6B,C).

To explore the prognostic role of this risk model, patients with PRAD were divided into low- and high-risk groups based on the risk scores. The risk plot depicted a reliable prognostic value of the risk score ( Figure 6D). Survival analysis demonstrated better PFS in patients with a low risk score relative to patients with a high risk score ( Figure 6E; *P*<0.0001). Subsequently, we performed an ROC curve analysis. We assessed the area under the curve for 1-year, 3-year, and 5-year PFS, which indicated that the model has better predictive power for survival outcomes ( Figure 6F). To assess the validity of this risk model, we further validated the external dataset using GEO microarray data (GSE21034; *n*=140). The results were the same as those of the TCGA cohort. The survival analysis demonstrated a better BCR-free survival status in patients with a low-risk score relative to patients with a high-risk score ( Figure 6G; *P*=0.0057). Additionally, we performed an ROC curve analysis. The areas under the curve for 1-year, 3-year, and 5-year BCR-free survival were 0.72, 0.64, and 0.66 ( Figure 6H), respectively, indicating that the risk model was well validated in the two separate datasets.

### Patients with high and low risk scores exhibit different clinical features

**Figure 7:**
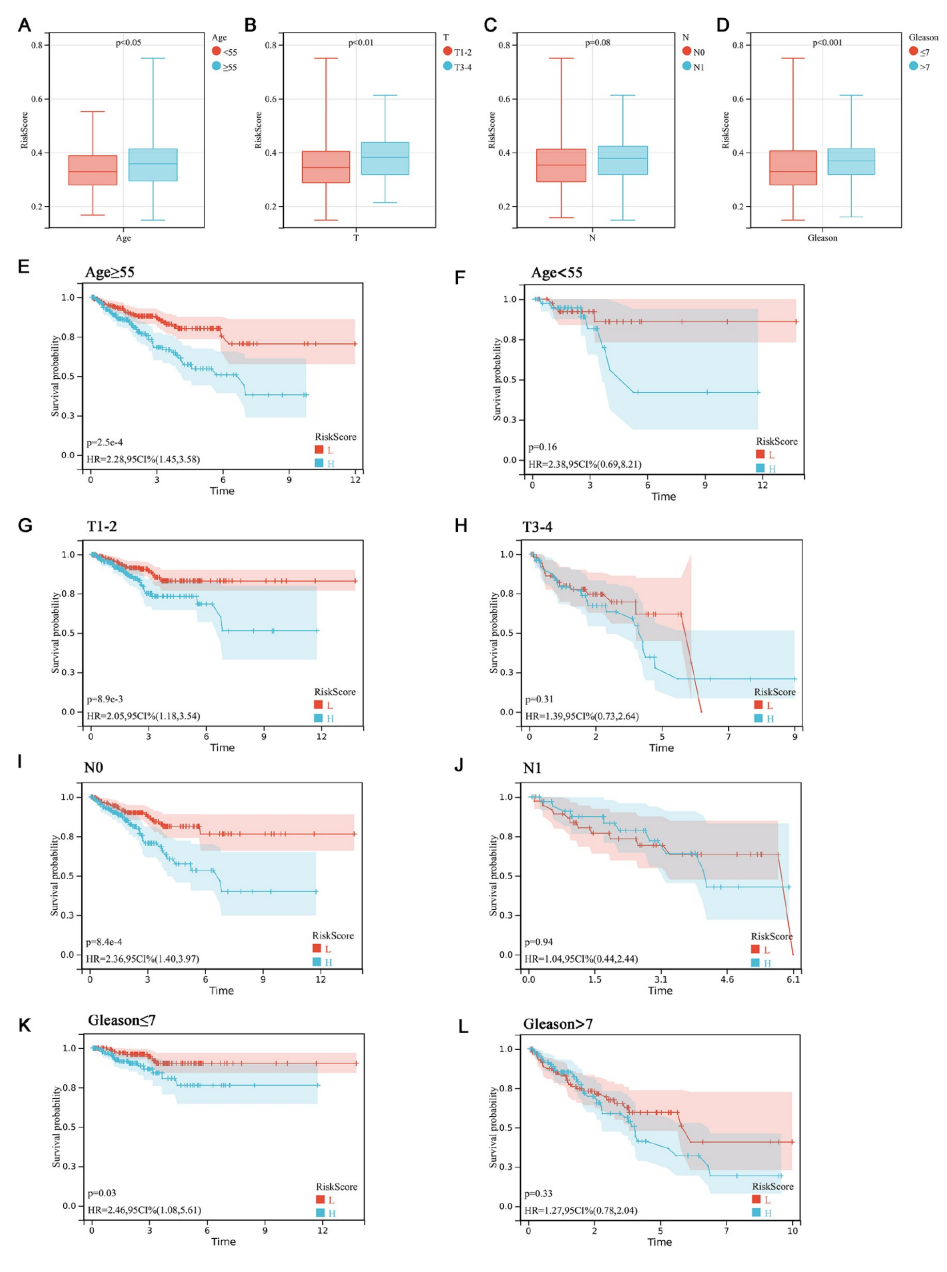
Figure 7 Risk score-based clinicopathological characteristic stratification in TCGA-PRAD (A‒D) The distribution of risk scores in different clinicopathological characteristics in the TCGA-PRAD sets. (E‒L) Kaplan-Meier curves of PFS differences stratified by age, T stage, N stage, or Gleason score between the low- and high-risk groups in the TCGA-PRAD sets. P<0.05 was defined as statistically significant.

To further evaluate the constructed risk model, we first explored the association between risk scores and clinical characteristics. Our analysis revealed that patients with different ages ( Figure 7A), T stages ( Figure 7B), and Gleason scores ( Figure 7D) showed significant differences in their risk scores ( *P*<0.05), indicating a partial association between the risk scores and clinical characteristics. Furthermore, when patients were grouped according to the above clinical characteristics, it was found that the risk model showed effective predictive performance for the “age≥55”, “T1-2”, “N0”, and “Gleason≤7” groups; moreover, patients with lower risk scores had a better prognosis ( Figure 7E–L). This suggests that the risk model has a better predictive effect for the low and medium clinical-stage groups with patients above 55 years of age.

In addition, univariate/multivariate Cox regression analyses revealed that the constructed risk model is an independent predictive marker for the prognosis of patients with PRAD ( Supplementary Tables S1 and S2). Additionally, we constructed a nomogram integrating the risk model and clinical features to more intuitively and accurately predict the outcome of patients with PRAD ( Supplementary Figure S2). The results showed that the nomogram could systematically predict the 1-, 3-, and 5-year PFS/BCR-free survival of patients with PRAD.

### IHC of representative proteins

Gene expression analysis showed that the HRs of the eight m7G regulatory genes were greater than 1 ( *P*<0.05), indicating their potential for promoting PCa. Among them, *NCBP2* and *EIF4A1* had the most significant prognostic value ( Figure 6A). Therefore, we chose NCBP2 and EIF4A1 for IHC validation.

**Figure 8:**
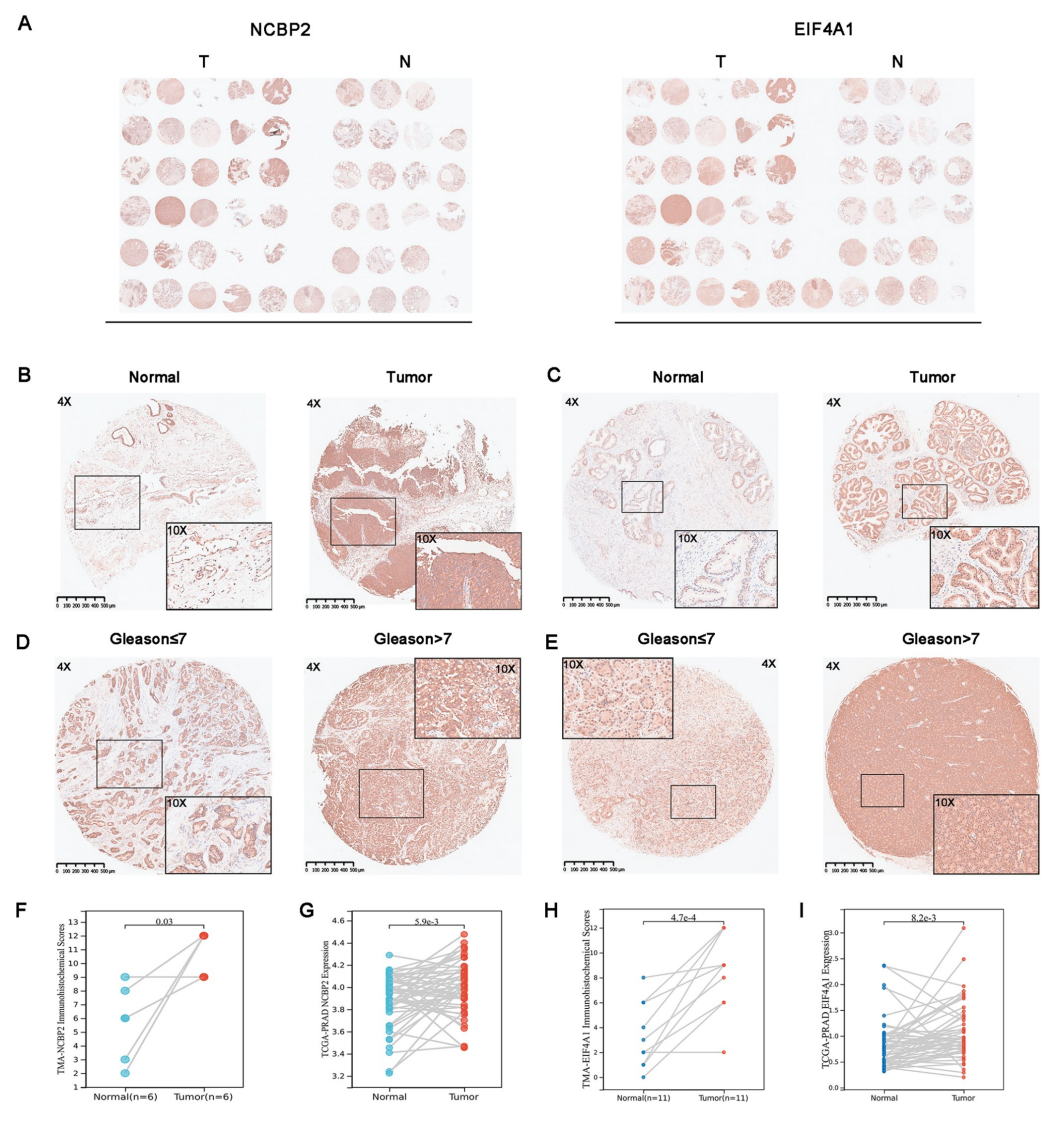
Figure 8 Immunohistochemistry staining of representative protein (A) The left panel shows the expression of NCBP2 in TMA tissue, and the right panel represents the expression of EIF4A1 in TMA tissue, respectively. (B,C) Representative images of immunohistochemical staining of NCBP2 (B) and EIF4A1 (C) in tumor and adjacent tissues, respectively. (D,E) Immunohistochemical plots of NCBP2 (D) and EIF4A1 (E) characteristics of different Gleason grade groups. (F,H) A paired t-test was performed on all paired samples of TMA samples. (G,I) Paired t-test for mRNA of TCGA paired samples.

To validate the protein expression of the risk gene signature, we performed IHC analysis on NCBP2 and EIF4A1 using PRAD pathologic specimens obtained from laparoscopic radical prostatectomy (LRP). The characteristics of the enrolled patients are illustrated in Supplementary Table S3. The NCBP2 and EIF4A1 protein expression levels in tumor tissues were significantly higher than those in the corresponding adjacent tissues ( Figure 8A‒C,F,H), which is consistent with the mRNA expression patterns of *NCBP2* and *EIF4A1* for TCGA-PRAD ( Figure 8G,I). We also analyzed the protein expressions of NCBP2 and EIF4A1 in different Gleason grade groups, which showed that with an increase in Gleason score, the protein expressions of NCBP2 and EIF4A1 were also increased significantly ( Figure 8D,E). This result is consistent with the mRNA expression pattern of *NCBP2* and *EIF4A1* for TCGA-PRAD ( Supplementary Figure S3), reinforcing the predictive role of *NCBP2* and *EIF4A1* expression for malignant PCa.

## Discussion

Similar to DNA and protein, RNA can be post-transcriptionally modified [24]. The modification process is usually catalyzed by specialized and highly conserved enzymatic mechanisms; there are approximately 170 different methods of modification, and the impairment of this modification process can yield a range of diseases [ 6, 25] . Modifications in mRNA contribute to the post-transcriptional regulation of mRNA fate [26]. Although N6-methyladenosine is the most common modification in mammalian mRNA, other modifications, including N1-methyladenosine, 5-methylcytosine, m7G, N4-acetylcytidine, ribose methylation, and pseudouridine, are present in mRNA and play functional roles [ 10, 26, 27] . Among the above RNA modifications, m7G has been gaining attention in the past few years, mainly in the cap position of mRNA molecules, tRNA variable loop, and eukaryotic 18S rRNA [ 14, 15, 28] . Our study further focused on the analysis of m7G modifications. Recent intensive studies on RNA modification have shown that RNA methylation modifications exert a wide range of critical functions, ranging from early development and viral infection to cancer [ 26, 29] . *METTL1* and *WDR4* are m7G modification regulators. In the AKT signaling pathway, the METTL1-WDR4 complex is directly phosphorylated by AKT and is engaged in the invasion and metastasis of various cancer types [30]. In lung cancer cells, *METTL1* knockdown in A549 cells and Caco-2 cells resulted in significantly enriched m7G-containing miRNAs, including miR-92(3p), let-7(5p), and miR-125(5p) [31]. In hepatocellular carcinoma, m7G regulatory proteins regulate oncogenic mRNA translation and cancer progression [32]. High *METTL1* expression is associated with poor patient prognosis and leads to downregulation of the expression of the tumor suppressor PTEN [33]. In addition, recent studies have shown that m7G regulator-mediated tRNA modifications promote the expressions of growth-promoting proteins by reconfiguring the mRNA “translationome,” thereby driving oncogenic transformation [34]. However, previously, the role of m7G RNA regulatory proteins in PCa remained unexplored. Therefore, there is a need for a holistic study on m7G RNA modification regulators. Given the BCR in a large number of patients with PCa, potential therapeutic targets are urgently needed. However, the two methods of analyzing RNA modification [liquid chromatography followed by mass spectrometry and high-throughput sequencing-based approaches] have some key limitations and are expensive. To ensure efficient use of research investments and to improve the accuracy of the study, we first analyzed different molecular subtypes and constructed risk models using two public databases (TCGA and GEO) and validated them using 46 pathological specimens obtained from LRP.

We selected 18 important m7G-related DEGs, and the gene‒gene interaction network of the 18 DEGs indicated that their functions were mainly enriched in RNA binding, methyltransferase activity, and translation regulation. Subsequently, the TCGA cohort was divided into two clusters, and we performed functional enrichment analysis and protein‒protein interaction analysis. We found that DEGs were mainly enriched in the biological processes of tumor motility and migration, tumorigenesis, tumor development, and the classical pathways of tumor pathology. DEGs between the two groups of clusters were correlated with the dysregulation of the immune system and metabolism. In addition, GSEA was enriched in pathways associated with tumorigenesis and immune regulation-related pathways. It is well known that the tumor microenvironment, which consists of non-cellular and cellular components, including immune cells, endothelial cells, and fibroblasts [35], plays an important role in tumor initiation and development [36]. In this context, immune cells are the pivotal components [37]. Based on the above pathway analyses of the two clusters, it is evident that immune dysregulation plays a crucial role in defining their differences. We further analyzed the immune cell infiltration of different clusters based on different algorithms. The results demonstrated that patients in cluster 1 had significantly higher stromal and immune cell scores ( *P*<0.05), such as B cells, T cells, and macrophages. Studies have shown that a low immune score and immune status are associated with unfavorable prognosis in osteosarcoma [ 38, 39] . Furthermore, our subsequent survival analysis revealed that although cluster 2 (the low immune score cluster) showed lower 5- and 10-year survival rates ( Supplementary Figure S4), there was no significant difference between the two clusters, possibly because PCa is an inert tumor with slow disease progression and fewer deaths.

Through univariate Cox regression analysis of 18 DEGs, we obtained eight genes significantly associated with PFS time. LASSO Cox regression analysis selected two critical genes, and we constructed a risk model for prognosis. Despite the limitations of this retrospective research, our risk model was validated using two independent cohorts. The survival analysis and ROC curve of the TCGA-PRAD dataset and the external validation dataset (GSE21034) illustrated that our risk model effectively stratifies patient outcomes, even in research cohorts with different sequencing methods and different populations. Therefore, our risk model is a reliable prognostic indicator of PCa. In addition, the risk score has a significant relationship with many clinicopathological features. The risk scores increase with age, T stage, and Gleason score, suggesting that the risk model can effectively predict tumor progression while accounting for the importance of the risk score in predicting PCa outcomes. Currently, most tumor risk models are based on clinicopathological features such as clinical symptoms, pathology, and histological scores. However, in recent years, many researchers have explored the application of genomics in tumor risk prediction. In particular, the role of transcripts should not be overlooked in constructing risk models, as they may be suitable elements to establish tumor risk models. Moreover, the genes in our risk model have been previously reported to be related to cancer. For instance, *EIF4A1* is a protein-coding gene that can interact with *PDCD4* to inhibit helicase activity and translation [ 40, 41] . Diseases associated with *EIF4A1* include pancreatic cancer [ 42, 43] , primary acute myeloid leukemia [44], and gastric cancer [45]. Among its related pathways are the mTOR signaling pathway and translational control [ 45, 46] .

To further clarify *NCBP2* and *EIF4A1* expression in PCa, we performed an IHC analysis of NCBP2 and EIF4A1. Our results showed that NCBP2 and EIF4A1 proteins were significantly highly expressed in tumor tissue and that the protein expression of *NCBP2* and *EIF4A1* increased significantly with an increasing Gleason score. The IHC results of the TMA further clarified the predictive role of NCBP2 and EIF4A1 in malignant PCa.

Despite the potential clinical relevance of our results, there are some limitations. First, the clinical characteristics extracted from TCGA and GEO databases are limited and incomplete. Second, it is unclear how environmental factors or treatments such as chemotherapy, radiotherapy, and targeted drug therapy affect the identified gene signatures. The m7G regulatory gene signature and derived cut-off value were constructed based on RNA-seq data. Therefore, the process was prone to bias and error.

In summary, we analyzed the expression of m7G RNA modification regulators in PCa based on TCGA and GEO databases and distinguished two clustered subgroups with significant differences in the immune microenvironment based on m7G-related DEGs. In addition, we identified and validated a reliable risk prediction model of m7G RNA modification regulators in PCa. Most importantly, by generating TMA-IHC from 46 collected clinical surgical samples, we identified NCBP2 and EIF4A1 as potential therapeutic targets in patients with PCa.

## Availability of Data and Materials

The datasets and R codes used during the current study are available on Jianguoyun ( https://www.jianguoyun.com/p/DV2DNfIQ4OfPChjYhMMEIAA).

The datasets analyzed during this study are available on the UCSC Xena browser ( http://xena.ucsc.edu/), GEO database (GSE21034; http://www.ncbi.nlm.nih.gov/geo), and cBioPortal for Cancer Genomics ( https://www.cbioportal.org/).

## Supporting information

421Supplementary

The_clinical_information_of_the_patients

## Supplementary Data

Supplementary data is available at *Acta Biochimica et Biophysica Sinica* online.
