# Supplementary material for: Expression pattern and prognostic value of key regulators for N7-methylguanosine RNA modification in prostate cancer: m7G RNA modification in PCa
Source: Acta Biochim Biophys Sin (Shanghai). 2023 Feb 22;55(4):561–73. doi: 10.3724/abbs.2023017 (PMC10195147; doi:10.3724/abbs.2023017)
Supplement: 421Supplementary [file 421Supplementary.pdf]

Supplementary Figures

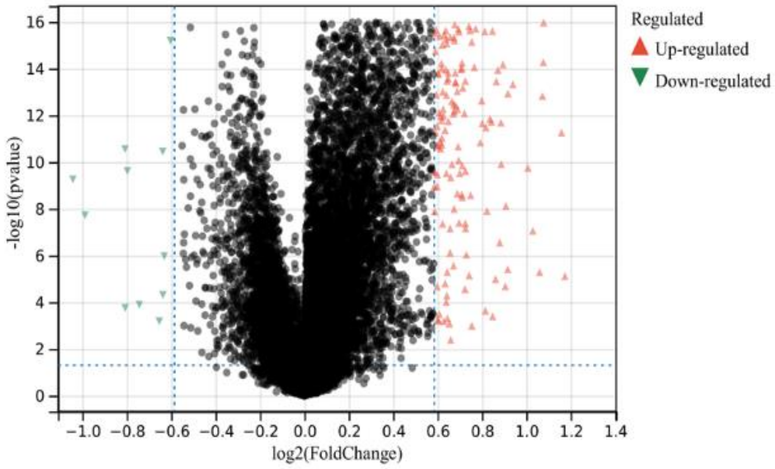

Supplementary Figure S1. Volcano plots of cluster subgroups We used R software package "Limma" for differential analysis to obtain differential genes between different cluster subgroups. A total of 443 DEGs were detected, of which 432 genes were up-regulated and 12 genes were down-regulated.

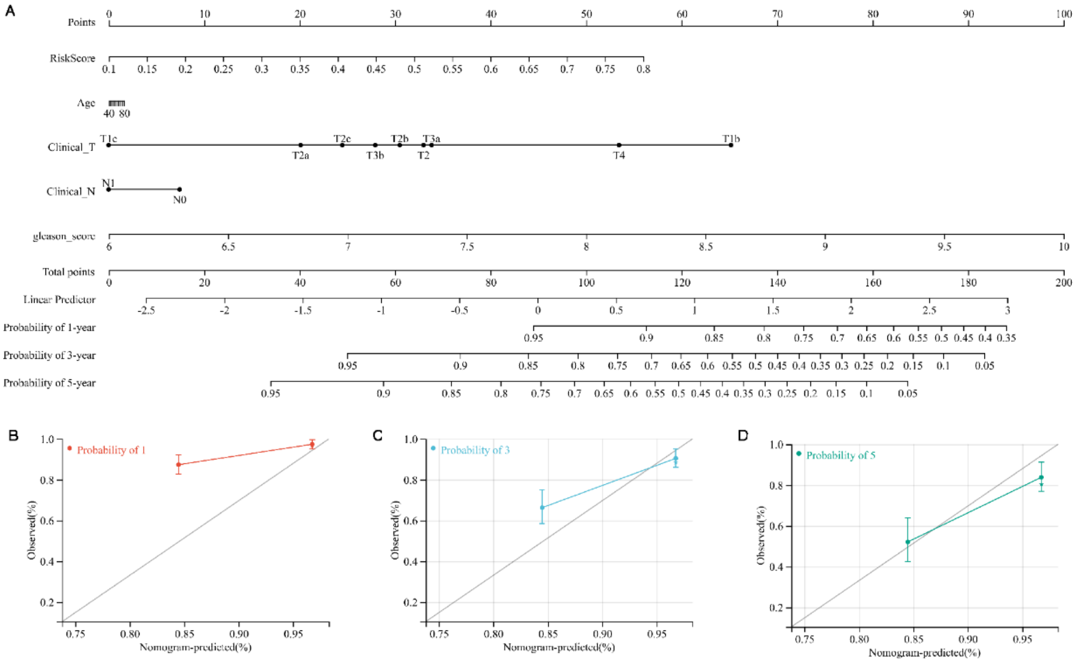

Supplementary Figure S2. Construction and calibration of nomogram (A) Nomogram integrating risk models and clinical characteristics. We used the R software package "RMS" to integrate the data of survival time, survival status and five characteristics, and established a Nomogram by Cox method to evaluate the prognostic significance of these characteristics in PRAD samples. (B-D) The

calibration curves of the constructed models. The overall C-index of the model was 0.740445251289891, 95%CI (0.687042822614385-0.793847679965397), and *P*-value = 1.09687159400959e-18.

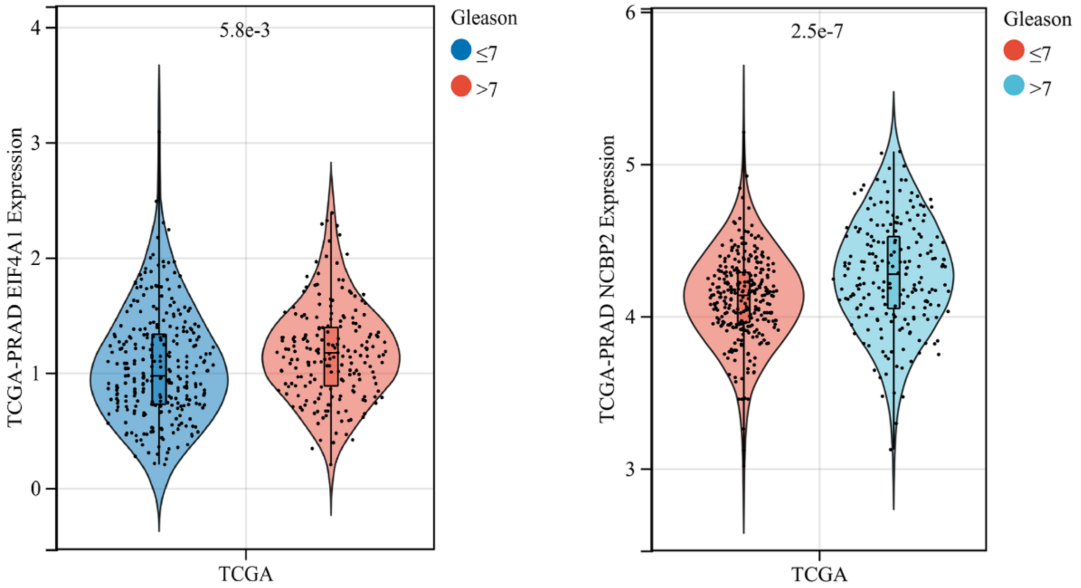

Supplementary Figure S3. Analysis of EIF4A1 and NCBP2 expression in TAGA-PRAD There were significant differences in *EIF4A1* and *NCBP2* mRNA expression levels between two Gleason score groups of TCGA.

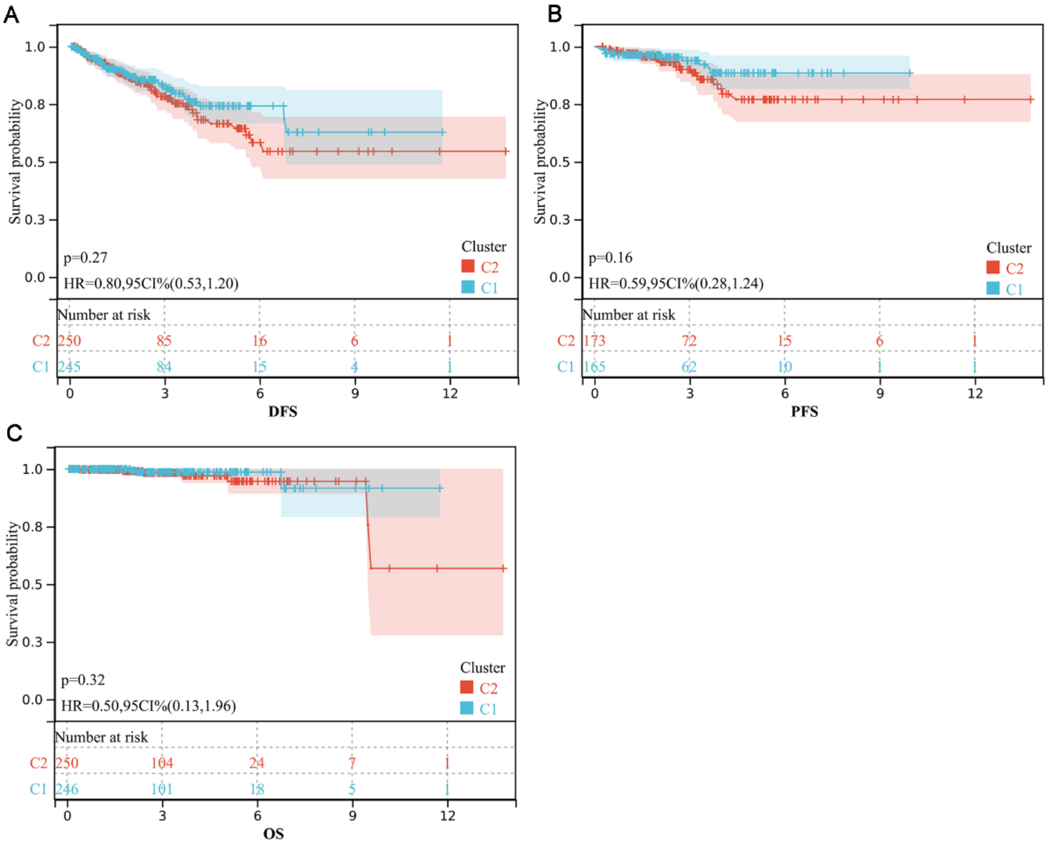

Supplementary Figure S4. Survival analysis of two cluster subgroups (A) DFS, (B) PFS, and (D) OS showed lower 5- and 10-year survival rates for cluster 2 (low immune score cluster), but there was no significant difference, probably because PCa is an inert tumor with slow disease progression and fewer deaths.

Supplementary Tables

Supplementary Table S1. Univariate analysis of risk score and characteristics

univariateCOX	coefficient	HR	HR(95%CI) (lower)	HR(95%CI) (upper)	*P*-value
RiskScore	3.605	36.779	4.434	305.113	0.001
Age	0.021	1.022	0.989	1.055	0.192
Clinical_T	0.679	1.972	1.497	2.596	0.000
Clinical_N	0.616	1.851	1.135	3.021	0.014
gleason_score	0.827	2.287	1.809	2.89	0.000

HR, hazard ratio; CI, confidence interval.

Supplementary Table S2. Multivariate analysis of risk score and characteristics

multivariateCOX	coefficient	HR	HR(95%CI) (lower)	HR(95%CI) (upper)	*P*-value
RiskScore	2.469	11.814	1.038	134.45	0.047
Age	0.003	1.003	0.971	1.036	0.869
Clinical_T	0.368	1.445	1.096	1.906	0.009
Clinical_N	-0.235	0.791	0.469	1.333	0.378
gleason_score	0.764	2.147	1.661	2.776	0.000

HR, hazard ratio; CI, confidence interval.

Supplementary Table S3. Clinical features and immunohistochemical scores (IHS) of patients with prostate cancer in the tissue microarray

Sample	Age	Gleason	EIF4A1-IHS	NCBP2-IHS
PCA1	70	9	6	9
PCA2	58	7	6	6
PCA3	73	10	9	12
PCA4	82	9	12	8
PCA5	67	7	9	9
PCA6	64	7	12	12
PCA7	83	0	12	12
PCA8	73	7	6	9
PCA9	62	9	9	9
PCA10	73	7	9	9
PCA11	67	7	9	8
PCA12	74	8	12	12
PCA13	74	10	12	8
PCA14	66	8	2	2
PCA15	67	9	6	6
PCA16	63	9	6	9
PCA17	67	7	9	9
PCA18	66	9	9	8
PCA19	76	7	12	12
PCA20	79	9	9	9
PCA21	61	7	6	1
PCA22	62	9	12	12
PCA23	69	9	3	4
PCA24	57	6	8	8
PCA25	67	9	6	0
PCA26	71	8	12	9

Supplementary Table S4. Clinical characteristics of patients with prostate adenocarcinoma (The Cancer Genome Atlas)

Characteristics	Low risk (N=189)	High risk (N=306)	Total (N=495)
**Age**			
Mean±SD	60.42±6.90	61.40±6.78	61.03±6.84
Median[min-max]	61.00 [44.00,77.00]	62.00 [41.00,78.00]	61.00 [41.00,78.00]
**cT**			
T1-2	159 (32.32%)	221 (44.92%)	380 (77.24%)
T3-4	30 (6.10%)	82 (16.67%)	112 (22.76%)
**cN**			
N0	130 (30.81%)	214 (50.71%)	344 (81.52%)
N1	21 (4.98%)	57 (13.51%)	78 (18.48%)
**Gleason**			
>7	55 (11.11%)	149 (30.10%)	204 (41.21%)
≤7	134 (27.07%)	157 (31.72%)	291 (58.79%)
**Sex**			
MALE	189 (38.18%)	306 (61.82%)	495 (100.00%)
**Histological_type**			
Prostate Adenocarcinoma Acinar Type	186 (37.58%)	294 (59.39%)	480 (96.97%)
Prostate Adenocarcinoma, Other Subtype	3 (0.61%)	12 (2.42%)	15 (3.03%)
