# Supplementary material for: Expression pattern and prognostic value of key regulators for N7-methylguanosine RNA modification in prostate cancer: m7G RNA modification in PCa
Source: Acta Biochim Biophys Sin (Shanghai). 2023 Feb 22;55(4):561–73. doi: 10.3724/abbs.2023017 (PMC10195147; doi:10.3724/abbs.2023017)
Supplement: The_clinical_information_of_the_patients [file The_clinical_information_of_the_patients.pdf]

The clinical inform

Sample_ID	SEX	AGE	pathologic_T	pathologic_N
PCA1	MALE	70	T4	N0
PCA2	MALE	58	T2c	N0
PCA3	MALE	73	T3b	N0
PCA4	MALE	82	T4	N1
PCA5	MALE	67	T2a	N0
PCA6	MALE	64	T3b	N0
PCA7	MALE	83	/	/
PCA8	MALE	73	T2	N0
PCA9	MALE	62	T4	N1
PCA10	MALE	73	/	/
PCA11	MALE	67	T2a	N0
PCA12	MALE	74	T3b	N1
PCA13	MALE	74	T3a	N0
PCA14	MALE	66	T2b	N0
PCA15	MALE	67	T3a	N0
PCA16	MALE	63	T3b	N0
PCA17	MALE	67	T2b	N0
PCA18	MALE	66	T4	N1
PCA19	MALE	76	T3b	N0
PCA20	MALE	79	T3	N0
PCA21	MALE	61	T3b	N0
PCA22	MALE	62	T3a	N0
PCA23	MALE	69	T2c	N0
PCA24	MALE	57	T2a	N0
PCA25	MALE	67	T2b	N0
PCA26	MALE	71	T2	N0
PCA27	MALE	65	T2	N0
PCA28	MALE	68	T2	N0
PCA29	MALE	55	T2	N0
PCA30	MALE	72	T2	N0
PCA31	MALE	62	T2c	N0
PCA32	MALE	86	T2a	N0
PCA33	MALE	56	T2	N0
PCA34	MALE	59	/	/
PCA35	MALE	65	T3b	N0

ation of the patients

pathologic_M	Histological type	Gleason grade	WHO/ISU
M0	Prostate Adenocarcinoma Acinar Type	9	5
M0	Prostate Adenocarcinoma Acinar Type	7	2
M0	Prostate Adenocarcinoma Acinar Type	10	5
M0	Prostate Adenocarcinoma Acinar Type	9	5
M0	Prostate Adenocarcinoma Acinar Type	7	2
M0	Prostate Adenocarcinoma Acinar Type	7	3
/	Prostate Adenocarcinoma Acinar Type	/	/
M0	Prostate Adenocarcinoma Acinar Type	7	2
M1	Prostate Adenocarcinoma Acinar Type	9	5
/	Prostate Adenocarcinoma Acinar Type	7	/
M0	Prostate Adenocarcinoma Acinar Type	7	2
M0	Prostate Adenocarcinoma Acinar Type	8	5
M0	Prostate Adenocarcinoma Acinar Type	10	5
M0	Prostate Adenocarcinoma Acinar Type	8	4
M0	Prostate Adenocarcinoma Acinar Type	9	5
M0	Prostate Adenocarcinoma Acinar Type	9	5
M0	Prostate Adenocarcinoma Acinar Type	7	3
M0	Prostate Adenocarcinoma Acinar Type	9	5
M0	Prostate Adenocarcinoma Acinar Type	7	3
M0	Prostate Adenocarcinoma Acinar Type	9	5
M0	Prostate Adenocarcinoma Acinar Type	7	3
M0	Prostate Adenocarcinoma Acinar Type	9	5
M0	Prostate Adenocarcinoma Acinar Type	9	5
M0	Prostate Adenocarcinoma Acinar Type	6	1
M0	Prostate Adenocarcinoma Acinar Type	9	5
M0	Prostate Adenocarcinoma Acinar Type	8	4
M0	Prostate Adenocarcinoma Acinar Type	7	2
M0	Prostate Adenocarcinoma Acinar Type	6	1
M0	Prostate Adenocarcinoma Acinar Type	9	5
M0	Prostate Adenocarcinoma Acinar Type	6	1
M0	Prostate Adenocarcinoma Acinar Type	6	1
M0	Prostate Adenocarcinoma Acinar Type	7	3
M0	Prostate Adenocarcinoma Acinar Type	6	1
/	Prostate Adenocarcinoma Acinar Type	10	5
M0	Prostate Adenocarcinoma Acinar Type	8	4
